# Sequential treatment for diabetic foot ulcers in aortic dissection patients: a case report

**DOI:** 10.3389/fendo.2025.1635004

**Published:** 2025-08-21

**Authors:** Huan Liang, Pin Deng, Lili Yang, Yusong Yuan, Xiaofang Ding

**Affiliations:** ^1^ Beijing Longfu Hospital, Beijing, China; ^2^ Institute of Basic Theory of Traditional Chinese Medicine, China Academy of Chinese Medical Sciences, Beijing, China; ^3^ Department of Trauma and Orthopaedics, China-Japan Friendship Hospital, Beijing, China; ^4^ Department of Orthopedics, Beijing Anzhen Hospital, Capital Medical University, Beijing, China

**Keywords:** diabetic foot ulcer (DFU), antibiotic-loaded bone cement (ALBC), aortic dissection (AD), case report, transverse tibial bone transport

## Abstract

Diabetic foot ulcers (DFUs) represent a prevalent complication of diabetes, with a lifetime risk ranging from 15% to 25% among diabetic patients. Research indicates that anticoagulation plays a crucial role in the management of newly diagnosed cases of diabetic lower extremity atherosclerotic obliterative disease. However, in the present case, the patient developed dry gangrene in both toes after receiving vasodilator drugs during an emergency intervention for sudden aortic dissection. Given that traditional treatments, such as anticoagulation and transfemoral amputation, may not be appropriate for every case, it is imperative to explore alternative therapeutic options. This case report presents pioneering clinical evidence of a tri-therapeutic strategy integrating biomechanical conditioning transverse tibial bone transport (TTBT), Traditional Chinese Medicine Compress Therapy (TCM-CT), and antibiotic-loaded bone cement (ALBC) for diabetic foot ulcer regeneration. This innovative paradigm demonstrates concurrent activation of mechanobiological signaling pathways, phytochemical bioactivity, and sustained antimicrobial protection, achieving complete epithelialization in refractory neuropathic wounds.

## Introduction

Diabetic foot ulcers (DFU) are among the most painful and severe chronic complications affecting elderly diabetic patients. Epidemiological data indicate that approximately 537 million individuals worldwide are living with diabetes (1, 2), and between 19% and 34% of these individuals will experience DFU (3) at some point in their lives.This prevalence is on the rise, suggesting that annually, between 9.1 million and 26.1 million new cases of DFU occur globally. Furthermore, studies have shown that approximately 5% to 8% of diabetic patients may require amputation surgery, which significantly impacts their quality of life (4). It has been reported that approximately 10% of diabetic patients undergo limb amputation due to vascular lesions (5). Stenotic or sclerotic lesions of the peripheral arteries lead to inadequate blood flow, causing the blood to become hypercoagulable and resulting in severe ischemia of the limb (6). This condition complicates wound healing, ultimately leading to the formation of a diabetic foot ulcer (DFU). The patient described in this report had a history of type 2 diabetes mellitus for over 20 years and was resuscitated due to sudden aortic dissection. During this event, the patient’s heart rate and blood pressure were tightly controlled, and the use of anticoagulants was contraindicated. The patient presented with bilateral color changes and was subsequently diagnosed with bilateral diabetic foot ulcers (DFU). During the rescue process, cardiologists administered high doses of vasodilators to maintain blood flow to vital organs and tissues, including the heart and lungs. Due to the self-hardening and narrowing of the peripheral arteries, insufficient blood flow in these arteries resulted in severe ischemia in both lower limbs. Diabetes mellitus is a major risk factor for cardiovascular disease. In patients with type 2 diabetes, chronic hyperglycemia, vascular endothelial inflammation, and systemic immune impairment collectively accelerate the progression of lower limb atherosclerotic gangrene. This pathological process evolves continuously, culminating in diabetic foot ulcers (DFUs). In clinical practice, these patients often fail to achieve complete remission and face a generally poor prognosis. This is particularly evident when complications such as lower limb arterial occlusion arise, which partially explains the high rates of mortality and amputation among diabetic foot patients in China (7). Therefore, developing affordable and effective treatment protocols is crucial to reduce mortality and amputation rates among diabetic foot patients with lower extremity atherosclerotic disease. This case represents the first documented instance of bilateral pedal gangrene developing after vasodilator therapy for aortic dissection in a diabetic patient. While DFUs affect 15-25% of diabetics, the co-occurrence of acute aortic catastrophe with subsequent iatrogenic limb ischemia constitutes an extremely rare (<0.1% reported) clinical scenario, necessitating paradigm-shifting therapeutic approaches.

## Case presentation

### Chief complaints

We report the case of a 56-year-old male patient with a history of type 2 diabetes mellitus diagnosed at the age of 35. In 2023, he experienced a sudden onset of aortic dissection. During the treatment of this condition, he suddenly developed bipedal DFU, which involved the tendons in the soles and toes of both feet.

### History of present illness

The patient had a Gangrene of both feet for one month, aggravated and painful for one week.

### Physical examination

The physical examination upon admission revealed a body temperature of 36.5°C, a pulse rate of 80 bpm, a respiratory rate of 18 breaths/min, and a blood pressure of 105/64 mmHg. The patient was thin, with a body mass index of 19.56 kg/m². Notably, there was dry gangrene present on both feet, characterized by large, thick black crusts covering the soles and toes, with blurred borders. On the left plantar surface, yellow necrotic tissue was observed at the base of an ulcer measuring approximately 4 cm by 7 cm. A small amount of fat covering was visible locally, along with minimal secretion. The inner side of the big toe on the back of the left foot exhibited redness and swelling, with an elevated local skin temperature. Additionally, the middle base of the right plantar ulcer appeared purulent, although there was no significant exudation. A small amount of synovial membrane was visible locally, along with exposure of the plantar fascia.

### Laboratory examinations

The patient was admitted to the hospital for laboratory tests and the results of the blood tests were as follows:

-White blood cell (WBC) count, 10.91 × 10^9^/l;
-Neutrophil count, 8.49 × 10^9^/l;
-c-reactive protein (CRP), 97 mg/l;
-Albumin, 30g/l;
-Urine sugar, 2++ (qualitative urine protein test);
-Fasting blood glucose, 6.4 mmol/l;
-HbA1c, 8.8%;
-Haemoglobin concentration 94 g/L
-Calcitoninogen, 0.03 ng/mL

## Final diagnosis

We report the case of a 56-year-old male patient with a history of type 2 diabetes mellitus, diagnosed at the age of 35. In 2023, he experienced a sudden onset of aortic dissection. During the treatment for this condition, he developed acute bipedal diabetic foot ulcers (DFUs) affecting the musculotendinous tendons in the soles and toes of both feet. There are more than 10 classification methods for diabetic foot, such as Texas, Meggitt-Wagner, WIfI, and SINBAD. These classification methodologies exhibit variations in their conceptual frameworks and clinical applications, with each approach demonstrating unique advantages and limitations (8, 9). the Texas classification is a grading system that integrates ulcer depth, infection, and ischemia severity. This classification is typically applied to diabetic foot ulcer patients complicated by infection or ischemia. Its advantage lies in enabling dynamic assessment of therapeutic response in diabetic foot patients; but it has the disadvantage of lacking evaluation of diabetic neuropathy. The patient in this case report was assessed and diagnosed using the Texas classification system.The patient in this case report exhibited marked bilateral diabetic gangrene, with ulcers extending to tendon structures, exposed plantar fascia, and severe limb ischemia. Consequently, the DFUs of this patient were rated as diabetic foot Texas Classification Grade 3, Stage D.

## Treatment

**Figure 1:**
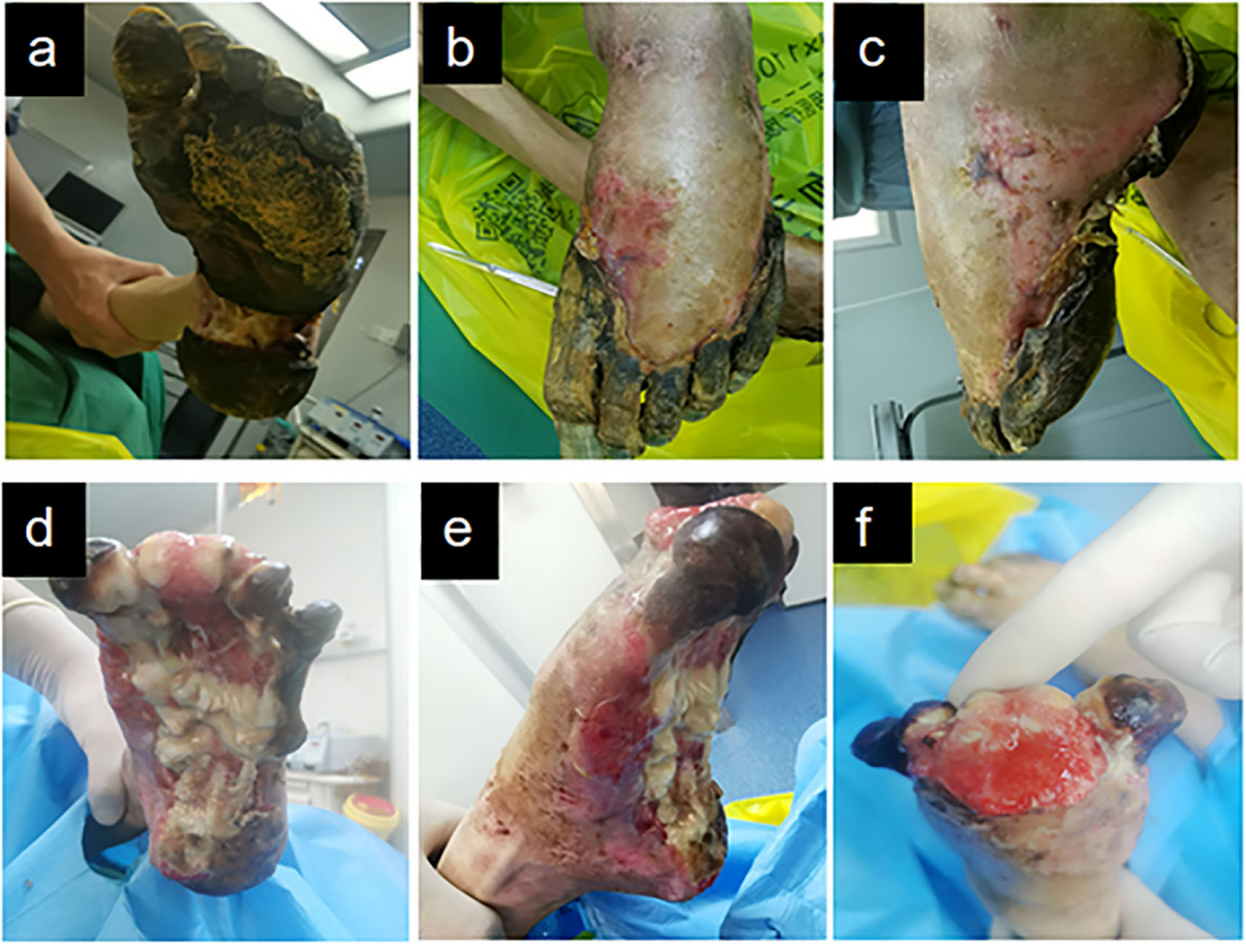
Left diabetic foot ulcer involving skin, muscle, and bone structures at initial presentation. **(a)** Necrotic margin on plantar surface. **(b)** Necrotic margin on dorsal surface. **(c)** Lateral view of the foot. **(d-f)** Initial debridement and drainage of the left foot performed following admission.

**Figure 2:**
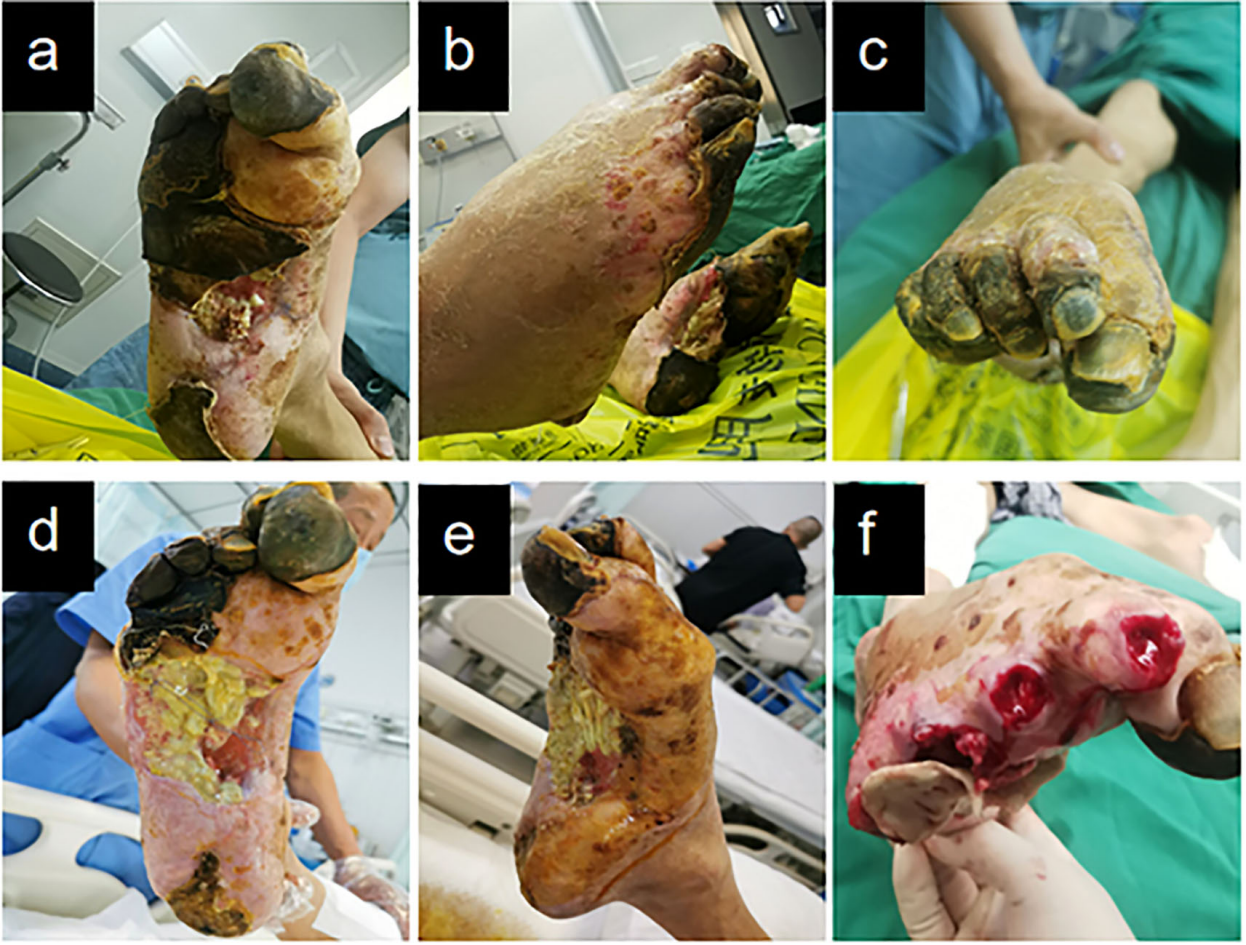
Right diabetic foot ulcer involving skin, muscle, and osseous structures at initial presentation. **(a)** Necrotic zone demarcation on plantar aspect. **(b)** Lateral foot view. **(c)** Necrotic zone demarcation on dorsal aspect. **(d–f)** Initial debridement and drainage of the right foot performed during hospitalization.

At the initial visit (**Figures 1a–c**), sharp debridement was conducted using scalpels, curets, and forceps following a decline in the patient’s inflammatory indicators. The thick black crust on the surface and the surrounding crust around the wound on the left foot were removed (**Figures 1d–f**). Upon removal of the hard crust, a significant amount of yellow purulent tissue became visible. The scalpel was then employed to further nibble and debride the wound, gently scraping the tissue until slight bleeding was observed at the edge of the skin. The thick crust in the center of the right foot (**Figures 2a–c**) was excised using a scalpel. Upon removal of the crust, a significant amount of foul-smelling purulent tissue was revealed (**Figures 2d, e**). A surgical knife was then employed to carefully debride the wound until the underlying tissue at the edges exhibited slight bleeding (**Figure 2f**). Due to the relatively poor blood supply to the patient’s right heel (**Figure 2d**) and the hardness of the thick black crust, the patient was temporarily treated with a local wet compress using traditional Chinese medicine for the compress therapy. Traditional Chinese herbal compresses enhance local perfusion through combined pharmaco-thermal effects. This facilitates targeted drug delivery to the lesion site, achieving rapid peak local drug concentration. Consequently, the treatment effectively suppresses inflammatory exudation, accelerates exudate absorption, and promotes the elimination of pathological products, thereby significantly enhancing wound healing.

Following bilateral foot debridement, the wounds were dressed with functional alginate-based primary dressings, overlaid with polyhexamethylene biguanide (PHMB)-impregnated antimicrobial gauze under compression bandages. Dressings were renewed every 48 hours. Post-procedural standardized instructions—both written and verbal-were provided to the patient and spouse, detailing essential wound care protocols and training in clinical recognition of infection signs with corresponding emergency response algorithms.

**Figure 3:**
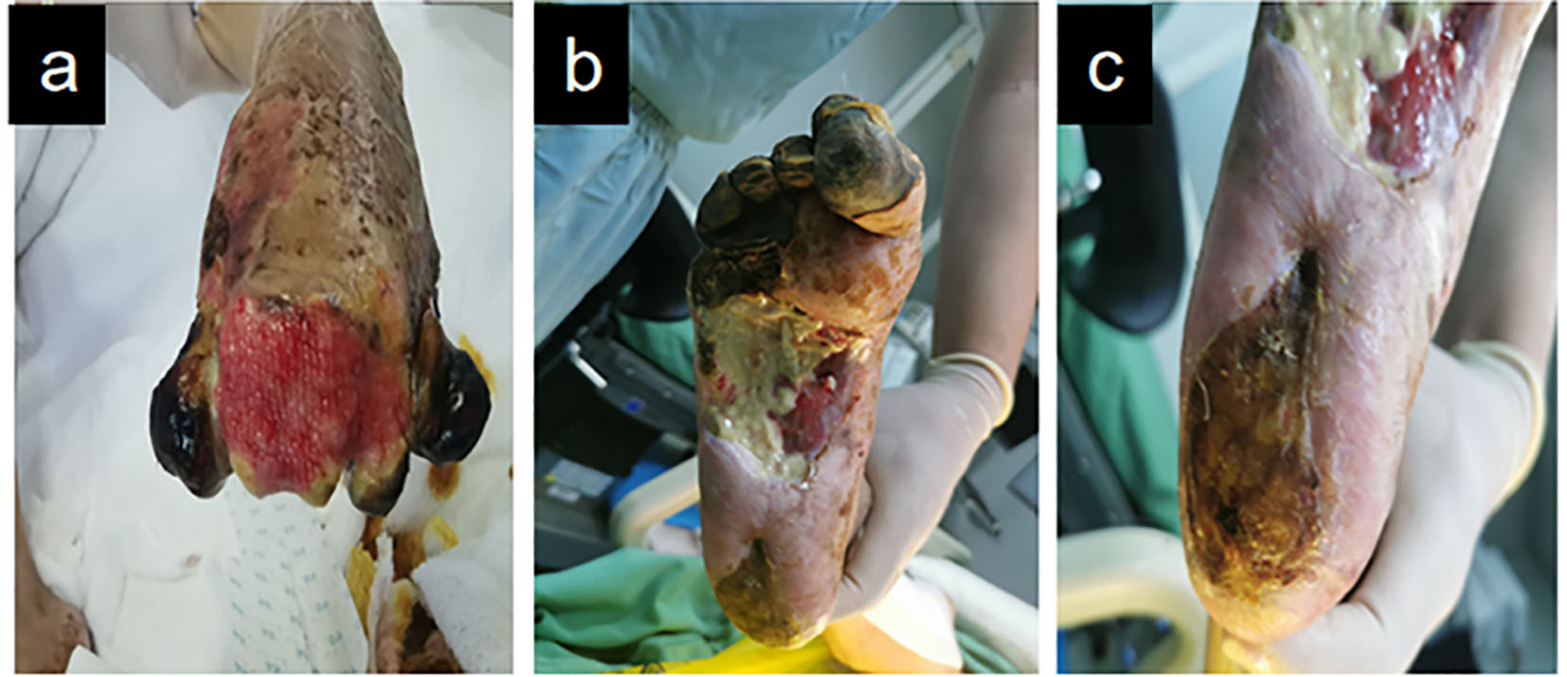
Demonstrates the improved wound condition of the patient’s foot one week following tibial bone transport and debridement procedures. **(a)** Dorsal forefoot wound (left). **(b)** Medial midfoot wound (right). **(c)** Plantar heel wound (right).

Given bilateral diabetic foot pathology and requisite prolonged bed confinement, the patient was instructed to perform active ankle dorsiflexion-plantarflexion exercises during recumbency to prevent deep vein thrombosis (DVT) in the lower extremities. For patients with diabetic foot conditions who are typically bedridden, dressing-changing devices should be employed to position their feet appropriately, thereby minimizing the risk of pressure ulcer formation. One week later, TTBT was initiated. Following Ilizarov’s tension-stress principle, a percutaneous low-energy corticotomy was performed on the medial tibia, creating a monofocal square bone segment while preserving periosteal integrity. Specialized distraction brackets were then applied to advance the bone segment at a controlled rate (typically 0.25-1 mm/day), inducing gradual tensile stress within the regenerate tissues. This procedure effectively stimulates the regeneration of the vascular system. The patient exhibited fresh granulation tissue growth on the dorsal aspect of the left toe, characterized by ruddy wounds (**Figure 3a**), as well as granulation tissue development at the toe end of the left foot, without significant local purulent tissue. The sore surface in the medial middle of the right foot remained stable (**Figure 3b**), with a slight covering of local adipose tissue, and the wound did not extend into the surrounding normal tissue. The right heel exhibited reduced eschar rigidity compared to baseline measurements, with viable tissue noted in the peri-wound area on clinical assessment (**Figure 3c**).

**Figure 4:**
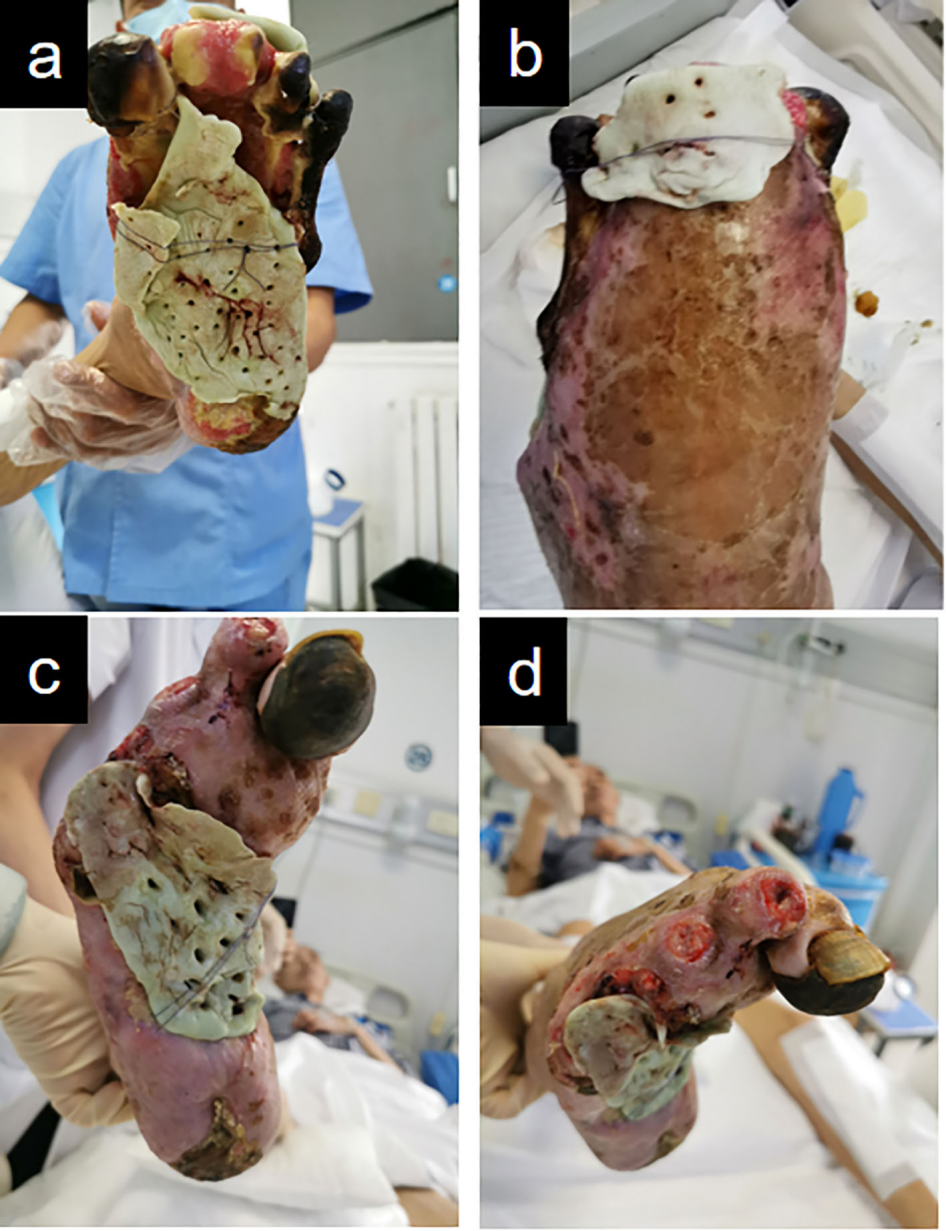
Illustrates the application of antibiotic-loaded bone cement for wound coverage on both feet. **(a, b)** Left foot. **(c, d)** Right foot.

Given that the patient’s wounds on both feet had persisted for over six months, he was referred for X-rays of both feet. The findings were inconclusive but indicated a potential diagnosis of osteomyelitis. The patient underwent weekly debridement and received alternate-day traditional Chinese herbal compress therapy. By postoperative week 4 (**Figure 4**), transverse tibial bone transport (TTBT) had been maintained for three weeks. Pathological and imaging evaluations ruled out osteomyelitis in both feet; however, wound cultures were positive for bacteria. To augment infection management, antibiotic-loaded bone cement (ALBC) was utilized to fill and cover bilateral foot wounds, secured locally with sutures. ALBC effectively fills wound cavities while continuously releasing antibiotics to inhibit infection and remove necrotic tissue (10). Furthermore, the induced membrane microenvironment generates various growth factors and recruits stem cells, thereby enhancing wound bed regeneration and maximizing therapeutic efficacy. The flexible duration of cement coverage provides an ideal foundation for secondary surgical repair. Dressings were changed thrice weekly, with scheduled debridement performed once per week.

**Figure 5:**
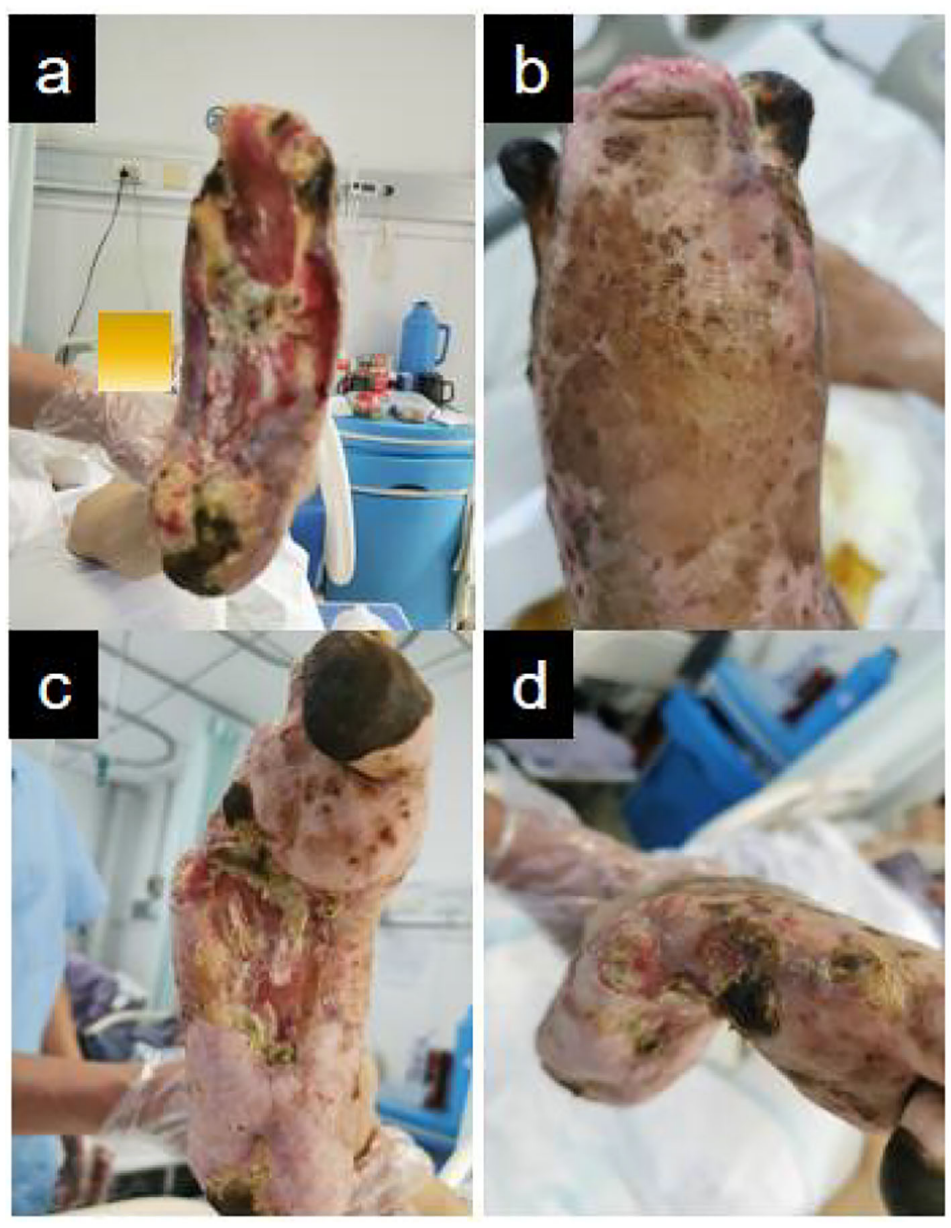
Shows that Antibiotic-loaded bone cement-mediated wound coverage with localized protective membrane formation and granulation tissue promotion. **(a, b)** Left foot. **(c, d)** Right foot.

At week 7 (**Figure 5**), the wound bed was clean with a mature neomembrane overlying the bone cement. Infected tissue volume substantially decreased, accompanied by nascent granulation tissue formation (**Figures 5a–d**). Debridement of the right distal toe exposed viable granulation tissue beneath the eschar following necrotic tissue removal (**Figure 5d**).

**Figure 6:**
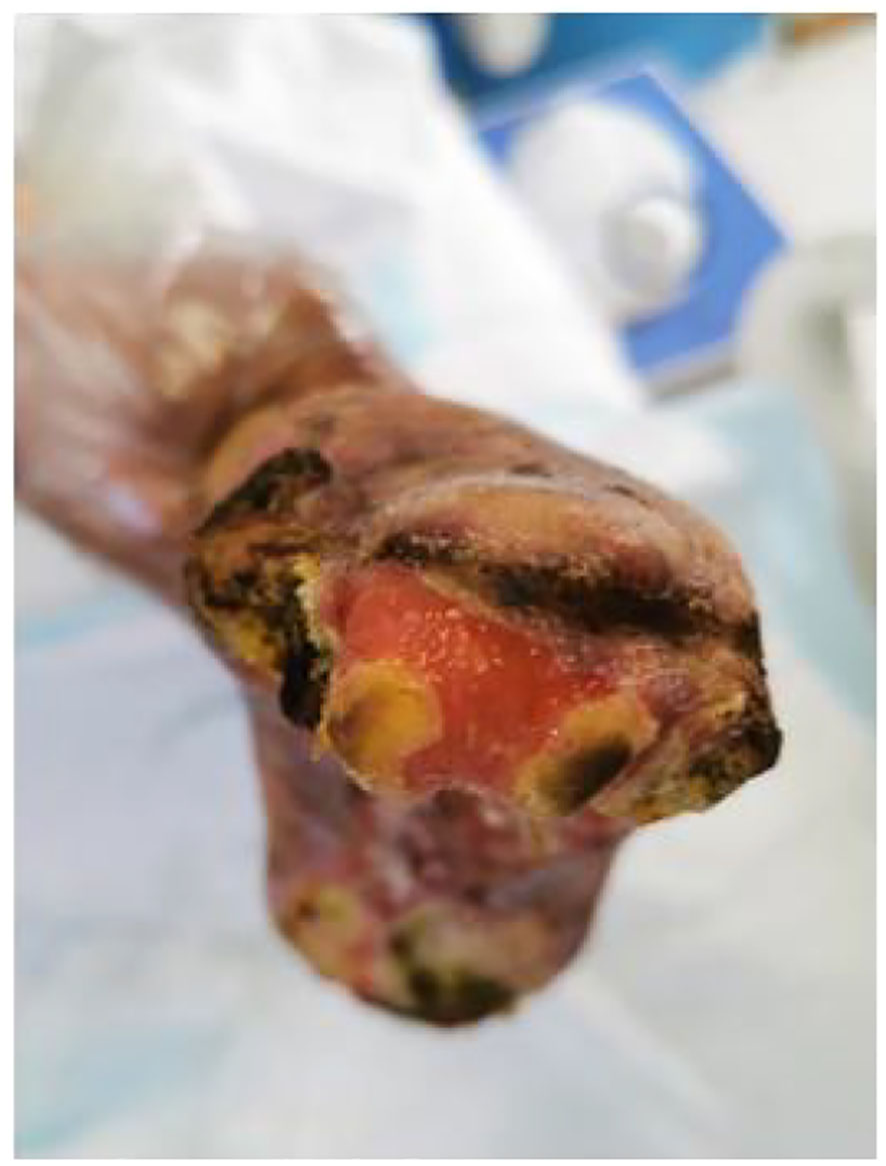
Clinical stabilization phase of left dorsal foot ulceration: Resolving inflammation and advancing epithelialization.

At week 10, the wound bed was packed with biofibrous dressing to maintain moisture balance and promote healing. The dorsal left toe exhibited a clean, dry surface with partial re-epithelialization (**Figure 6**).

**Figure 7:**
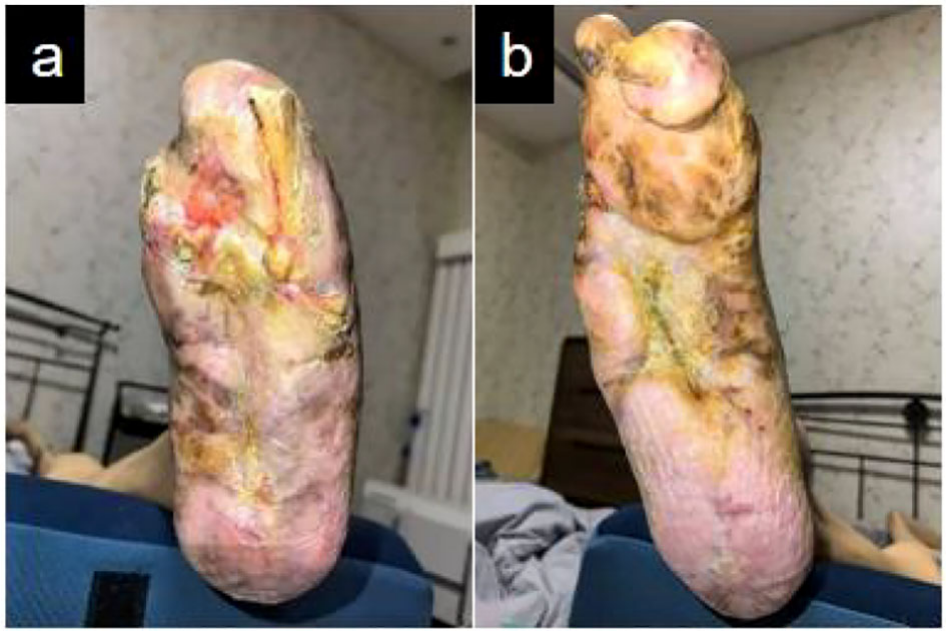
Complete wound closure in bilateral lower extremities: **(a)** Left foot with full epithelial restoration; **(b)** Right foot with complete epithelial healing.

At 18 weeks postoperatively (**Figure 7**), the patient underwent regular dressing changes in the outpatient clinic. Wound closure was successfully achieved in both feet, allowing the patient to wear simple slippers. Additionally, the clinician routinely examined the patient’s feet for any signs of redness or skin trauma.

**Figure 8:**
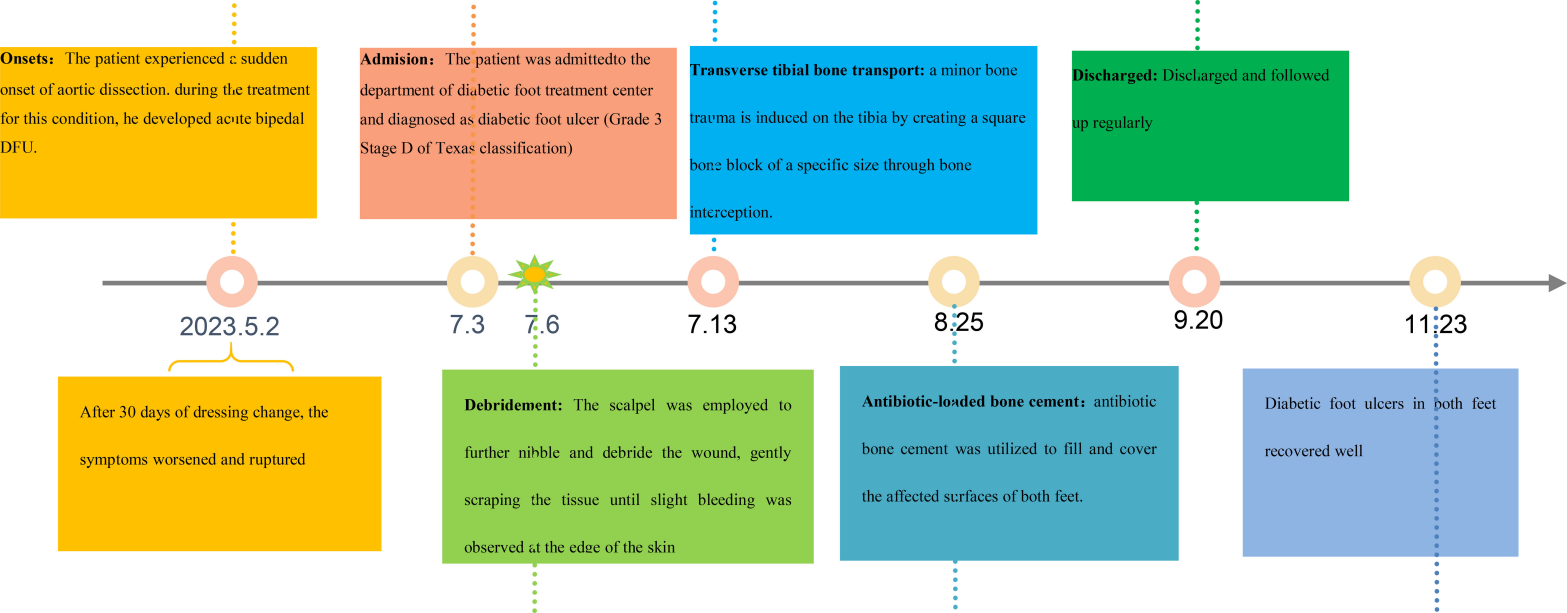
Timeline of the treatment process for a case from DFU onset to healing.

The patient underwent regular monitoring for one month after discharge, during which time the wound closure remained stable. Given the patient’s history of aortic dissection, it is recommended that the patient undergo multidisciplinary follow-up at the cardiology and podiatry outpatient clinics. The timeline of the entire treatment process is shown in **Figure 8**.

## Outcome and follow-up

**Figure 9:**
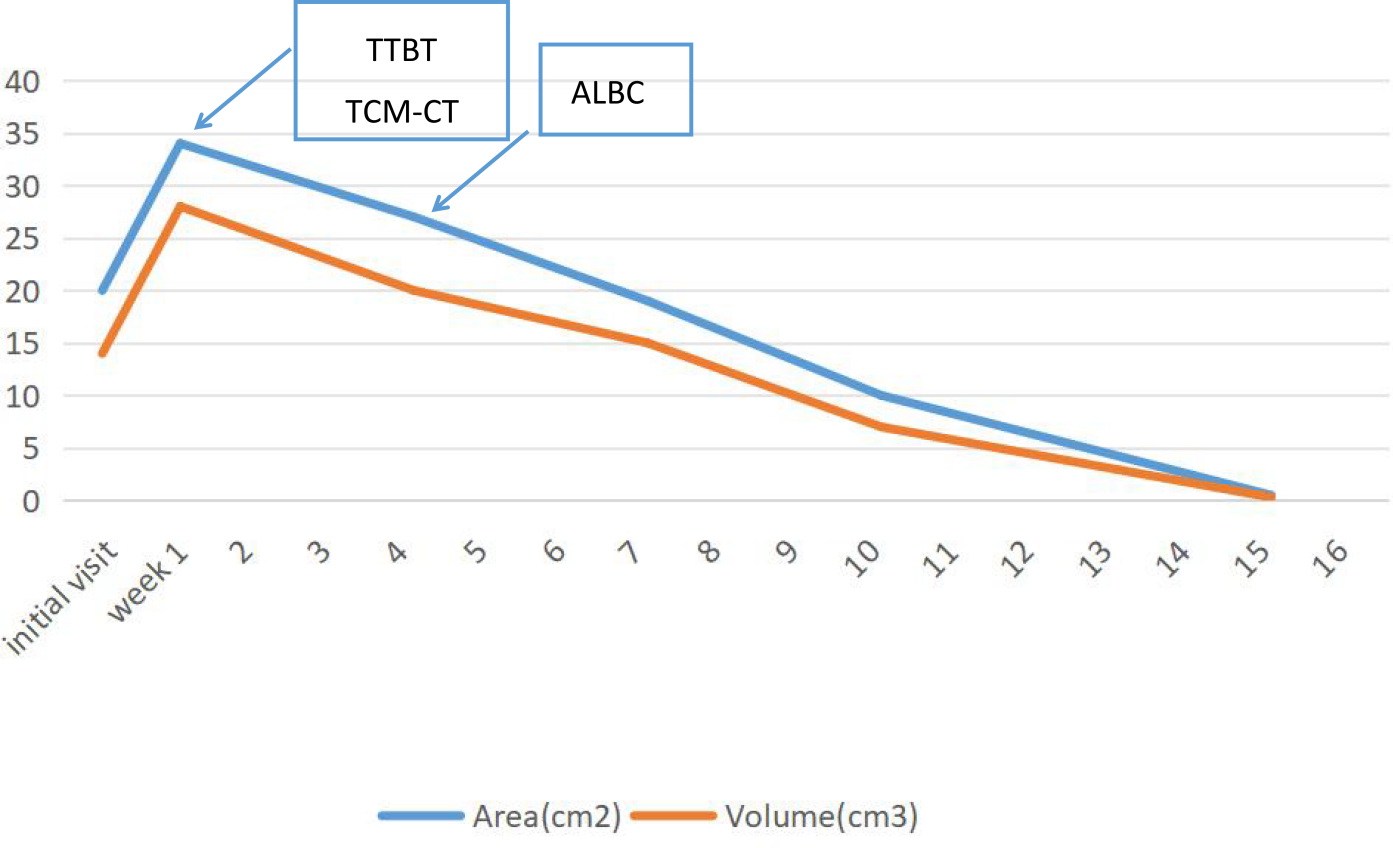
Area and wound measurements over time with transverse tibial bone transport (TTBT), Traditional Chinese Medicine Compress Therapy (TCM Compress Therapy), and antibiotic-loaded bone cement (ALBC) equivalent interventions indicated.

**Figure 9** illustrates the wound measurements of the patient throughout the treatment process. Upon initial presentation, the patient exhibited dry gangrene on both feet. Debridement significantly enlarged the wounds, which were characterized by the presence of yellow pus and necrotic tissue at their bases. One week later, ultrasonic debridement of the foot wound resulted in further enlargement. By week 4, there was a notable reduction in the amount of pus and slough tissue on the surface of the sores, with a 25% decrease in wound depth. Additionally, the wounds on both feet were covered with antibiotic bone cement. In the 7th week, following the application of antibiotic bone cement to the wounds, a local wound protective film was successfully induced, facilitating continued wound closure.

At week 10, the wound size on both feet had decreased compared to initial measurements. Following the patient’s discharge from the hospital, dressings were changed regularly in the outpatient department. Eighteen weeks later, the patient achieved 100% epithelialization of the wounds on both feet. During subsequent follow-up appointments, the patient’s wounds remained closed.

### Compliance with ethics guidelines

The authors received consent from the patient included in this case report.

## Discussion and conclusion

Risk factors for diabetic foot ulceration (DFU) include smoking, inadequate blood sugar control, peripheral neuropathy, foot malformations, and peripheral vascular disease. In this case, the patient has a smoking history exceeding 30 years, along with foot malformations and peripheral neuropathy. Additionally, he has endured type 2 diabetes for more than 20 years, during which blood glucose control posed significant challenges. However, following hospitalization, the patient’s blood glucose levels were effectively managed under the close supervision of endocrinologists within a multidisciplinary consultation framework.

The patient’s condition is quite complicated, with a high risk of diabetic foot ulcers (DFU) and potential amputation (11). Wu et al. (12) suggested that education may play a crucial role in reducing these risks. Both the patient and their spouse are university educators, which enables them to adhere strictly to medical advice. It is recommended that health guidance for the patient and their spouse, along with long-term follow-up via phone calls after discharge, constitutes an essential component of the comprehensive approach outlined in this case report. This strategy provides an improved opportunity for the patient to manage controllable risk factors effectively.

The treatment principles for diabetic foot ulcers (DFU) are widely recognized to include diabetes management, effective local wound care, infection control, decompression strategies, and the promotion of smooth blood circulation. In this case, all of these principles are being effectively addressed. The patient’s endocrinologist is closely monitoring blood sugar levels and assessing blood flow.Although the measured oxygen partial pressure in the left transcutaneous limb was 50 mmHg and in the right transcutaneous limb it was 60 mmHg, the blood and oxygen supply to both lower limbs appeared to be adequate. However, ultrasound and X-ray results of the blood vessels in the lower limbs indicated that the patient had severe segmental stenosis of the lower limb arteries. Consequently, the percutaneous oxygen partial pressure is deemed excessively high to accurately assess the degree of ischemia and hypoxia in the foot. In this instance, we executed bilateral tibial osteotomies to enhance blood supply to the distal regions of both lower extremities. The patient’s wound healing may be associated with neovascularization induced by tibial bone transport (Ilizarov technique) (13). This procedure utilizes a minimal surgical incision on the medial tibia, where a longitudinal periosteal incision is made (preserving the periosteum). Controlled mechanical stimulation is then applied to activate the growth of bone marrow and periosteal (inner and outer layer) cells. The periosteum, rich in microvessels and growth factors, provides a critical anatomical basis for effective diabetic foot ulcer (DFU) treatment through its cortical vessels and vascular network. Over a 15-day treatment cycle, the bone transport technique delivers sustained, controlled mechanical stimulation. This promotes local vascular regeneration and repair, converting mechanical energy into biological regenerative signals. The high-intensity mechanical stimulation effectively overcomes the inhibitory effects of the hyperglycemic environment on vascular regeneration, thereby establishing a significant mechanobiological foundation for diabetic foot treatment.

Throughout the wound treatment process, we adhered strictly to the TIME principle. During the initial assessment of the wound, we noted the presence of extensive necrotic tissue in the patient’s feet, prompting us to perform effective debridement. Following this, we selected appropriate antibiotics for anti-inflammatory treatment based on the results of the patient’s wound secretion culture. Once we confirmed the complete removal of necrotic tissue, we applied antibiotic bone cement to cover the affected foot’s wound, successfully inducing a protective film and promoting wound healing. In the middle and late stages of wound treatment, we apply biocellulose dressings to cover the local sore surface. This approach helps maintain moisture, regulates the absorption and evaporation of water, and creates a conducive environment for the migration of epithelial cells. Ultimately, complete epithelialization of the wounds on both feet was achieved. Currently available wound dressings cannot ensure rapid and effective healing of injured tissue. Among these, chronic diabetic wounds, which involve multiple diabetes-related complications (14), have no single dressing that can address all their issues. As345 a result, emerging nanotechnology is being widely explored to address these chronic complications. Nanocarrier technology offers novel breakthroughs in the treatment of diabetic foot ulcers (DFUs). By enhancing drug delivery efficiency, improving the wound microenvironment, and promoting tissue regeneration, this technology effectively addresses limitations of conventional therapies, such as poor drug stability, low bioavailability, and high susceptibility to infection (15). Core technologies include: Nanofiber dressings: Engineered via electrospinning to form porous barrier structures, enabling sustained drug release. For instance, chitosan-polyethylene oxide (PEO) nanofibers encapsulating terpinen-4-ol-loaded liposomes effectively inhibit drug-resistant bacteria and accelerate epithelial regeneration (16). Hydrogels: Provide a moist healing environment and can be loaded with multifunctional components to modulate inflammation, scavenge reactive oxygen species (ROS), and thereby accelerate wound healing. Liposomes: Their encapsulation capacity significantly enhances the solubility and stability of hydrophobic drugs (17). For example, curcumin-loaded flexible liposomes combined with polycaprolactone (PCL) form composites demonstrating superior antibacterial activity and biocompatibility compared to traditional dressings (18). Polymeric nanoparticles: Materials like poly(lactic-co-glycolic acid) (PLGA) nanoparticles function as drug carriers while also exhibiting immunomodulatory properties (19). Lipid-coated nanoparticles: Can induce antibody production and effectively combat multidrug-resistant bacteria. Research shows that different nanocarriers, with their unique properties, demonstrate multifunctionality in addressing specific issues related to chronic wounds (20).

We regularly perform debridement on patients’ wounds on a weekly basis. It is important to note that we employ a technique known as “Chinese Medicine Nibbling Debridement”, which adheres to the principles of working from the periphery to the center and from soft tissue to tougher tissue. This method involves the careful removal of non-viable cell debris in small amounts over multiple sessions, removing necrotic and hyperkeratotic tissue. During each debridement session, the edges of the wound should be beveled to promote the migration of epithelial cells. Additionally, adequate drainage must be provided for wounds with sinus tracts. Iodophor solution is used to clean the area before and after each procedure.

In the early stages, the patient exhibited significant clinical manifestations of infection. After ensuring the complete removal of necrotic tissue from the patient’s foot, the patient displayed considerable bone destruction in both feet. antibiotic bone cement was applied to cover the wound. This procedure, known as the Masquelet technique, not only protects exposed structures (tendons, bones, and soft tissues) but also induces a vascularized membrane. Critically, the bone cement gradually releases antibiotics, making topical delivery significantly more effective than intravenous administration for infection control (21).

In the 10th week of treatment, the patient’s wound bed was free of necrotic tissue, although significant depth remained. The application of a biological cellulose dressing continues to be recommended for filling local wounds. The biological cellulose dressing is characterized by its high water content and low mechanical properties, which contribute to maintaining a moist environment and providing hydration; furthermore, it does not adhere to the wound upon replacement. Research by GOKOO indicates that biological cellulose hydrogel dressings offer distinct advantages in promoting wound healing and epithelial reconstruction compared to other water colloid dressings (22, 23). This patient’s healing time exceeded the average duration reported in the literature for DFUs, with wound healing occurring over a period of 70 to 126 days. During treatment, we performed TTBT to enhance microcirculation in the lower limbs. While this procedure is effective, it is costly. Sheehan et al. (24) suggested that advanced therapies should be considered when patients are not progressing along a normal healing trajectory, particularly when the potential for expensive complications outweighs the treatment costs. Given the complexity and chronic nature of the wounds, we employed advanced therapies. This case successfully managed complex DFU (Texas 3D grade) using a combination of TTBT, TCM-CT, and ALBC, achieving complete wound healing with 100% epithelialization in both feet. The core advantage of this triple therapy lies in simultaneously addressing vascular ischemia, deep infection, and tissue regeneration.

In recent years, nanotechnology has demonstrated potential advantages in precise drug delivery and the rapid healing of complications (25–27). Future developments could include a ‘nanotechnology-enhanced integrated traditional Chinese and Western medicine therapy’. Examples include combining TTBT with nanobionic scaffolds to accelerate vascular regeneration and alleviate pain; In the future, we can integrate traditional Chinese medicine with nanocarrier systems to enhance the transdermal absorption rate of active ingredients for precise drug delivery; and developing nanocomposite bone cement to improve the efficiency of deep infection control and reduce the frequency of surgical replacements. “Simultaneously, non-invasive vascular imaging technology will be employed to observe in real time how TTBT dynamically improves lower limb microcirculation and to elucidate how these mechanical stress changes facilitate the targeted delivery of TCM-CT active ingredients while enhancing their therapeutic effects on ischemic tissue. This approach will provide direct visual evidence of the core synergistic mechanism underlying ‘mechanical force-guided drug targeted delivery,’ addressing the clinical need to alleviate ischemia-related complications. “During wound management, scheduled debridement was performed on both feet of the patient. However, due to compromised lower limb circulation and reduced immunity, the local wound site was prone to secondary infection. While antibiotic-loaded bone cement coverage can provide localized drug release, it suffers from issues such as burst release effect and incomplete eradication of drug-resistant bacteria. In contrast, antibacterial nanomaterials can be directly integrated into bone cement or dressings. This approach extends the antibacterial duration, promotes faster transition of the wound into the proliferative phase, and lowers the risk of recurrence.

Diabetic refractory wounds represent a major clinical challenge. Prolonged hyperglycemia induces angiopathy, neuropathy, and compromised immune function in patients. Collectively, these pathological changes facilitate pathogen colonization and infection, severely impeding the wound healing process. Notably, patients undergoing tibial bone transport surgery frequently develop skin necrosis due to local tissue thinning and compromised blood supply. Given the significant potential of nanotechnology in diabetic foot ulcer (DFU) treatment (28), our literature review reveals that polymer-based composite nanofiber dressings incorporating nanotechnology can effectively mimic the function of the human extracellular matrix (29), thereby providing an optimized microenvironment for tissue regeneration. For instance, studies have employed electrospinning to fabricate chitosan nanofiber membranes modified with the Chinese herbal compound baicalein for wound therapy. Results demonstrate that this dressing exhibits exceptional antioxidant and antibacterial activity while significantly accelerating wound healing (30).

In the context of the complex disease known as diabetic foot, achieving optimal treatment outcomes through a single discipline can often be challenging. Throughout this patient’s care, a multidisciplinary approach has proven to be essential for the successful management of patients with diabetic foot ulcers (DFU). Endocrinologists play a crucial role in managing the patient’s blood sugar levels, ensuring they remain within a reasonable range. Cardiologists focus on the patient’s aortic dissection, adjusting blood pressure and heart rate as necessary. Orthopedic surgeons are tasked with surgical interventions aimed at enhancing blood supply to the lower limbs, addressing foot muscle issues and ulcers, and regularly performing deformity corrections and ulcer debridement.

The advantage of multidisciplinary collaboration lies in its ability to thoroughly consider all facets of a patient’s condition, thereby enabling the formulation of a more precise and comprehensive treatment plan. Such an approach not only enhances treatment efficacy but also shortens the duration of treatment, ultimately reducing the financial burden and alleviating the physical and mental distress experienced by the patient.

## Data Availability

The original contributions presented in the study are included in the article/supplementary material. Further inquiries can be directed to the corresponding author.
